# 4-Hy­droxy-1,1′-bis­[(*S*)-1-phenyl­eth­yl]-5,5′,6,6′-tetra­hydro-3,4′-bipyridine-2,2′(1*H*,1′*H*)-dione

**DOI:** 10.1107/S1600536813004017

**Published:** 2013-02-20

**Authors:** Nancy Romero, Dino Gnecco, Joel Terán, Sylvain Bernès

**Affiliations:** a Universidad Juárez Autónoma de Tabasco, División Académica de Ciencias Básicas, Km. 1 carretera Cunduacán, Jalpa de Méndez AP 24, Cunduacán, Tabasco, Mexico; b Centro de Química, Benemerita Universidad Autónoma de Puebla, Edif. 103H Complejo de Ciencias, C.U., 72570 Puebla, Pue., Mexico; c Universidad Autónoma de Nuevo León, UANL, Facultad de Ciencias Químicas, Av. Universidad S/N, Ciudad Universitaria, San Nicolás de los Garza, Nuevo León CP 66451, Mexico

## Abstract

The title bis-piperidine, C_26_H_28_N_2_O_3_, was unexpectedly obtained *via* a dimerization mechanism promoted by acetic acid when performing the Dieckmann cyclization of a chiral amido ester. The *S*,*S* configuration was assigned by reference to the enanti­omerically pure starting material. In the mol­ecule, two core heterocycles are linked by a σ bond. One ring includes a keto–enol group, while the other presents an enone functionality. Both rings present a conformation inter­mediate between envelope and screw-boat, and the dihedral angle between the mean planes passing through the rings [48.9 (1)°] is large enough to avoid hindrance between ring substituents. The enol tautomeric form in one ring favors the formation of strong inter­molecular O—H⋯O=C hydrogen bonds. The resulting one-dimensional supra­molecular structure features single-stranded helices running along the 2_1_ screw axis parallel to [100].

## Related literature

For natural products having a bis-piperidine substructure, see: Gil *et al.* (1995 ▶); Torres *et al.* (2000 ▶); Matsunaga *et al.* (2004 ▶); Smith & Sulikowski (2010 ▶). For related structures of monocyclic piperidines, see: Didierjean *et al.* (2004 ▶); Romero *et al.* (2005 ▶). For the application of Dieckmann condensation in organic synthesis, see: Scheiber & Nemes (2008 ▶). For an example of self-condensation of a dione similar to that used for the synthesis of the title compound, see: Sugasawa & Oka (1954 ▶).
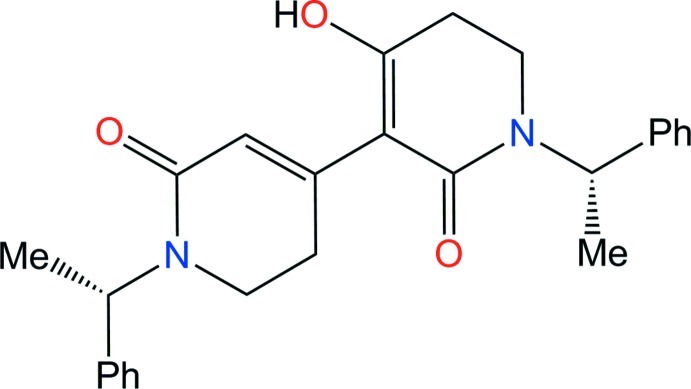


## Experimental

### 

#### Crystal data

C_26_H_28_N_2_O_3_
*M* *_r_* = 416.50
Orthorhombic, 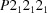

*a* = 9.6647 (13) Å
*b* = 9.7281 (10) Å
*c* = 23.684 (3) Å
*V* = 2226.7 (5) Å^3^
*Z* = 4
Mo *K*α radiation
μ = 0.08 mm^−1^
*T* = 296 K
0.60 × 0.60 × 0.08 mm

#### Data collection

Bruker P4 diffractometer
3173 measured reflections
2250 independent reflections
1843 reflections with *I* > 2σ(*I*)
*R*_int_ = 0.019
3 standard reflections every 97 reflections intensity decay: 1.5%

#### Refinement

*R*[*F* ^2^ > 2σ(*F* ^2^)] = 0.039
*wR*(*F* ^2^) = 0.095
*S* = 1.04
2250 reflections
286 parameters
H atoms treated by a mixture of independent and constrained refinement
Δρ_max_ = 0.19 e Å^−3^
Δρ_min_ = −0.15 e Å^−3^

### 

Data collection: *XSCANS* (Siemens, 1996 ▶); cell refinement: *XSCANS*; data reduction: *XSCANS*; program(s) used to solve structure: *SHELXS97* (Sheldrick, 2008 ▶); program(s) used to refine structure: *SHELXL97* (Sheldrick, 2008 ▶); molecular graphics: *Mercury* (Macrae *et al.*, 2008 ▶); software used to prepare material for publication: *SHELXL97*.

## Supplementary Material

Crystal structure: contains datablock(s) I, global. DOI: 10.1107/S1600536813004017/nr2039sup1.cif

Structure factors: contains datablock(s) I. DOI: 10.1107/S1600536813004017/nr2039Isup2.hkl

Supplementary material file. DOI: 10.1107/S1600536813004017/nr2039Isup3.cml

Additional supplementary materials:  crystallographic information; 3D view; checkCIF report

## Figures and Tables

**Table 1:** Hydrogen-bond geometry (Å, °)

*D*—H⋯*A*	*D*—H	H⋯*A*	*D*⋯*A*	*D*—H⋯*A*
O4—H4⋯O2′^i^	0.97 (4)	1.67 (4)	2.637 (3)	177 (4)

Symmetry code: (i) 
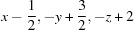
.

**Fig. 1.**
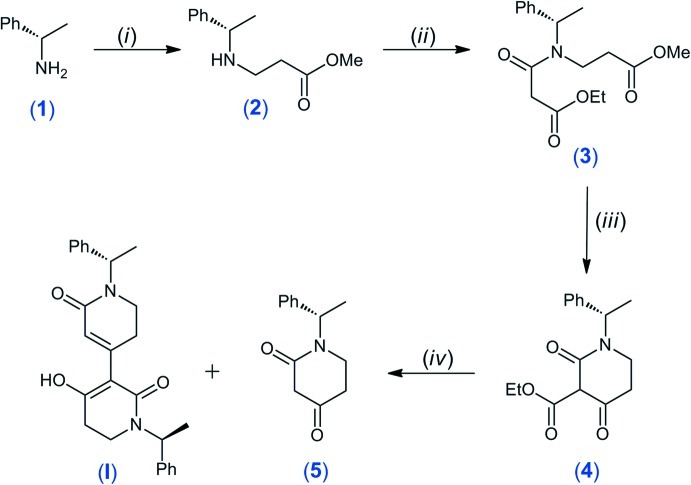
The synthesis of the title molecule, I. (i) Methyl acrylate, MeOH, 25 °C, 12 h. (ii) Diethyl malonate, reflux, 6 h. (iii) NaH, cyclohexane/toluene, reflux. (iv) AcOH/H2O (30%, v/v).

**Fig. 2.**
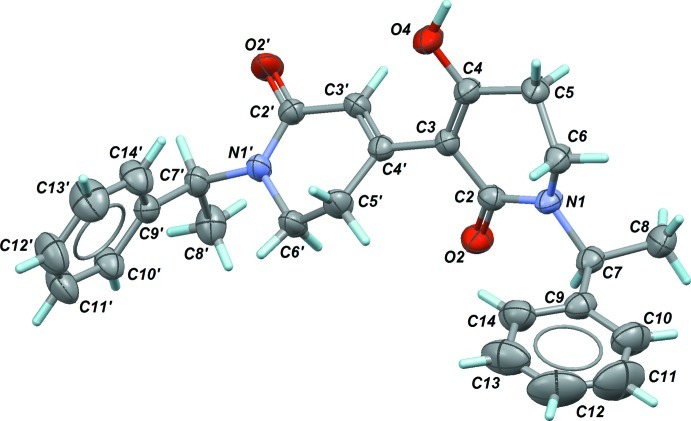
Molecular structure of the title compound, with 50% probability level displacement ellipsoids for non-H atoms.

**Fig. 3.**
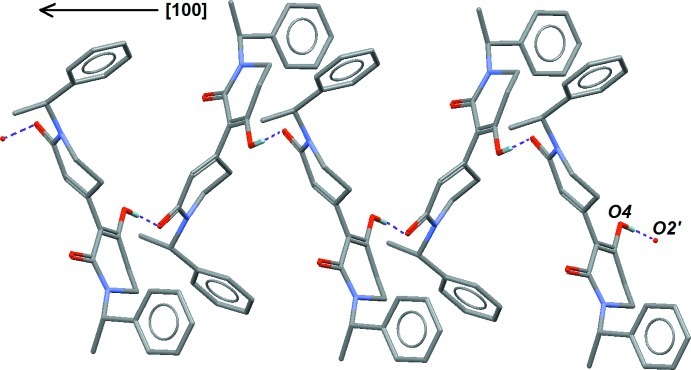
A chain of hydrogen-bonded molecules, along the screw axis parallel to [100].

## supplementary crystallographic information

### Comment

The title compound is a byproduct of the Dieckmann cyclization carried on the chiral amido ester 3 (Fig. 1). When concentrated acetic acid is used in the fourth synthetic step, a dimerization occurs during the decarboxylation process, affording the title molecule I as the major product, while the expected piperidine-2,4-dione 5 is obtained in low yield. This synthetic route for the preparation of this kind of piperidone derivatives is known to be successful in many cases (*e.g*. Scheiber & Nemes, 2008). However, it seems that the possible interference of secondary reactions like dimerization is poorly commented in the literature, probably because these reactions are seen as a trouble for the intended synthetic target. To the best of our knowledge, a single article clearly commented on this problem (Sugasawa & Oka, 1954). In this report, the authors added a note in the galley proofs, which is worth to quote in full: "in the course of the present work, we prepared *N*-benzyl-2,4-dioxopiperidine [···]. Our attempt to condense this ketone with ethyl cyanoacetate under Cope condition was not effected because this compound was found to undergo bimolecular self-condensation fairly rapidly, at a room temperature [···]. This tendency of the easy intermolecular self-condensation [of *N*-benzyl-2,4-dioxopiperidine] is so remarkable when compared with the stability of the corresponding 5-ethyl derivatives, which suffer no change when kept in a stoppered bottle at room temperature for a long time".

The synthesis of the title compound in good yield now confirms the observations done by Sugasawa & Oka 59 years ago.

The molecular structure of I is built up from one ring including a keto-enol group (ring N1/C2···C6) bonded to a ring with the enone functionality (ring N1'/C2'···C6', see Fig. 2). Both rings present a conformation intermediate between envelope and screw-boat, with Cremer parameters being θ = 118.1° and φ = 101.0° for the keto-enol ring, and θ = 60.5° and φ = 278.5° for the enone ring. The dihedral angle between mean planes passing through these heterocycles, 48.9 (1)°, is large enough to avoid hindrance between atoms O2 and O4 in the first ring and H atoms at C3' and C5' in the other ring. Heterocycles in I have indeed conformations close to those observed in monocyclic related compounds which were X-ray characterized (*e.g*. Didierjean *et al.*, 2004; Romero *et al.*, 2005). In the solid state, the enolic tautomer of I seems to be favored over the di-ketone because the presence of a donor OH group allows the formation of stabilizing intermolecular O—H···O═C hydrogen bonds in the crystal. These strong interactions generate a supramolecular structure based on single stranded helices running along the 2_1_ crystallographic screw axis in the [100] direction (Fig. 3).

The reported structure may be of interest in the field of natural products. It has been reported that the biosynthesis of some bis-piperidine alkaloids isolated from marine sponges, like halicyclamine A (Gil *et al.*, 1995) or haliclonacyclamine C (Smith & Sulikowski, 2010) could involve the dimerization of dihydropyridines. Other natural products of interest also share the title compound bis-piperidine scaffold, with additional points of cyclization between the piperidine rings (Torres *et al.*, 2000; Matsunaga *et al.*, 2004).

### Experimental

The synthesis is described in Fig. 1. A solution of 1 (41.2 mmol, 1 eq.) and methyl acrylate (49.6 mmol, 1.2 eq.) was stirred overnight at 298 K. The reaction mixture was concentrated under reduced pressure, and the crude purified by column chromatography (SiO_2_, CH_2_Cl_2_:MeOH, 97:3), to afford 2 as a colourless oil (98%). An amount of 2 (40.6 mmol, 1 eq.) was dissolved in diethyl malonate (40 ml) and the mixture refluxed until the reaction was complete (6 h). After concentration, the crude was chromatographed (Al_2_O_3_, *n*-hexane:AcOEt, 1:1), to afford 3, as a colourless oil (75%). A suspension of NaH (34.2 mmol, 2.5 eq.) in cyclohexane (100 ml) was refluxed for 20 min, and then, a solution of 3 (13.7 mmol, 1.1 eq. in 30 ml of anhydrous toluene) was added dropwise. After refluxing the mixture for 5 h, a solid was obtained, 4, which was filtered and dried in air. This solid was treated with acetic acid:water (30%, *v*/*v*) for the decarboxylation process. The mixture was refluxed until gas evolution stopped. After cooling down to 298 K, pH was adjusted to 7 with NaHCO_3_, and the mixture was washed with CH_2_Cl_2_ (3 × 50 ml). The organic phase was dried over Na_2_SO_4_, and concentrated. Compounds 5 and I were separated by column chromatography, (SiO_2_, CH_2_Cl_2_:MeOH, 95:5). The title compound I was obtained in 80% yield, and was recrystallized from AcOEt:*n*-hexane (1:1). m.p. = 444 K, [α]^20^_D_ = -172.5 (c=1, CH_2_Cl_2_). Compound 5, a colourless oil, was isolated in low yield (< 20%). Key NMR and IR data are given in the archived CIF.

### Refinement

All C-bound H atoms were placed in idealized positions and refined as riding to their carrier atoms, with bond lengths fixed to 0.93 (aromatic CH), 0.96 (methyl CH_3_), 0.97 (methylene CH_2_) or 0.98 Å (methine CH). Isotropic displacement parameters were calculated as *U*_iso_(H) = *xU*_eq_(carrier atom), with *x* = 1.5 (methyl groups) or *x* = 1.2 (other H atoms). H4 (hydroxyl group) was found in a difference map and refined with free coordinates and *U*_iso_(H4) = 1.5*U*_eq_(O4). The absolute configuration for C7 and C7' is based on the known configuration of the enantiomerically pure starting material, (*S*)-(–)-1-phenylethylamine, and 621 measured Friedel pairs were merged for refinement.

### Crystal data

**Table:**

C_26_H_28_N_2_O_3_	*D*_x_ = 1.242 Mg m^−^^3^
*M**_r_* = 416.50	Melting point: 444 K
Orthorhombic, *P*2_1_2_1_2_1_	Mo *K*α radiation, λ = 0.71073 Å
Hall symbol: P 2ac 2ab	Cell parameters from 62 reflections
*a* = 9.6647 (13) Å	θ = 4.7–12.0°
*b* = 9.7281 (10) Å	µ = 0.08 mm^−^^1^
*c* = 23.684 (3) Å	*T* = 296 K
*V* = 2226.7 (5) Å^3^	Plate, colourless
*Z* = 4	0.60 × 0.60 × 0.08 mm
*F*(000) = 888	

### Data collection

**Table:**

Bruker P4 diffractometer	*R*_int_ = 0.019
Radiation source: fine-focus sealed tube	θ_max_ = 25.0°, θ_min_ = 2.3°
Graphite monochromator	*h* = −11→2
ω scans	*k* = −11→1
3173 measured reflections	*l* = −1→28
2250 independent reflections	3 standard reflections every 97 reflections
1843 reflections with *I* > 2σ(*I*)	intensity decay: 1.5%

### Refinement

**Table:**

Refinement on *F*^2^	Secondary atom site location: difference Fourier map
Least-squares matrix: full	Hydrogen site location: inferred from neighbouring sites
*R*[*F*^2^ > 2σ(*F*^2^)] = 0.039	H atoms treated by a mixture of independent and constrained refinement
*wR*(*F*^2^) = 0.095	*w* = 1/[σ^2^(*F*_o_^2^) + (0.0483*P*)^2^ + 0.2404*P*] where *P* = (*F*_o_^2^ + 2*F*_c_^2^)/3
*S* = 1.04	(Δ/σ)_max_ < 0.001
2250 reflections	Δρ_max_ = 0.19 e Å^−^^3^
286 parameters	Δρ_min_ = −0.15 e Å^−^^3^
0 restraints	Extinction correction: *SHELXL97* (Sheldrick, 2008), Fc^*^=kFc[1+0.001xFc^2^λ^3^/sin(2θ)]^-1/4^
0 constraints	Extinction coefficient: 0.0098 (15)
Primary atom site location: structure-invariant direct methods	Absolute structure: Assigned from synthesis. Friedel pairs (621) were merged

### Fractional atomic coordinates and isotropic or equivalent isotropic displacement parameters (Å^2^)

**Table:**

	*x*	*y*	*z*	*U*_iso_*/*U*_eq_	
N1	0.6918 (2)	0.2061 (2)	0.96330 (9)	0.0342 (5)	
C2	0.7571 (3)	0.3060 (3)	0.93294 (10)	0.0324 (6)	
O2	0.8401 (2)	0.27815 (18)	0.89463 (8)	0.0451 (5)	
C3	0.7286 (3)	0.4508 (3)	0.94792 (10)	0.0330 (6)	
C4	0.6613 (3)	0.4827 (3)	0.99592 (11)	0.0360 (6)	
O4	0.6401 (2)	0.61396 (19)	1.01054 (8)	0.0515 (6)	
H4	0.591 (4)	0.629 (4)	1.0457 (14)	0.077*	
C5	0.6126 (3)	0.3712 (3)	1.03453 (11)	0.0397 (7)	
H5A	0.5315	0.4021	1.0551	0.048*	
H5B	0.6845	0.3498	1.0617	0.048*	
C6	0.5774 (3)	0.2438 (3)	1.00066 (12)	0.0395 (6)	
H6A	0.5579	0.1683	1.0262	0.047*	
H6B	0.4950	0.2608	0.9783	0.047*	
C7	0.7035 (3)	0.0612 (3)	0.94466 (11)	0.0364 (7)	
H7A	0.7807	0.0575	0.9179	0.044*	
C8	0.7425 (4)	−0.0316 (3)	0.99393 (13)	0.0523 (8)	
H8A	0.8233	0.0043	1.0123	0.078*	
H8B	0.7616	−0.1225	0.9802	0.078*	
H8C	0.6673	−0.0352	1.0204	0.078*	
C9	0.5745 (3)	0.0181 (3)	0.91220 (11)	0.0412 (7)	
C10	0.5143 (4)	−0.1103 (4)	0.91817 (15)	0.0656 (10)	
H10A	0.5525	−0.1738	0.9430	0.079*	
C11	0.3974 (5)	−0.1450 (5)	0.8874 (2)	0.0934 (15)	
H11A	0.3575	−0.2312	0.8920	0.112*	
C12	0.3405 (5)	−0.0541 (6)	0.8505 (2)	0.0936 (15)	
H12A	0.2614	−0.0779	0.8304	0.112*	
C13	0.3993 (4)	0.0718 (5)	0.84287 (17)	0.0778 (12)	
H13A	0.3616	0.1332	0.8170	0.093*	
C14	0.5151 (3)	0.1078 (4)	0.87365 (12)	0.0557 (9)	
H14A	0.5541	0.1942	0.8684	0.067*	
N1'	0.8952 (2)	0.7324 (2)	0.82498 (8)	0.0355 (5)	
C2'	0.9292 (3)	0.7510 (3)	0.87967 (10)	0.0340 (6)	
O2'	1.0011 (2)	0.8499 (2)	0.89480 (8)	0.0513 (6)	
C3'	0.8771 (3)	0.6493 (3)	0.92029 (10)	0.0370 (7)	
H3'A	0.9110	0.6518	0.9570	0.044*	
C4'	0.7837 (3)	0.5535 (3)	0.90715 (10)	0.0321 (6)	
C5'	0.7280 (3)	0.5523 (3)	0.84798 (11)	0.0430 (7)	
H5'A	0.7012	0.4593	0.8380	0.052*	
H5'B	0.6462	0.6098	0.8461	0.052*	
C6'	0.8320 (3)	0.6029 (3)	0.80695 (10)	0.0445 (7)	
H6'A	0.9038	0.5341	0.8024	0.053*	
H6'B	0.7879	0.6161	0.7706	0.053*	
C7'	0.9595 (3)	0.8177 (3)	0.78050 (10)	0.0380 (7)	
H7'A	0.9965	0.9002	0.7989	0.046*	
C8'	1.0818 (3)	0.7410 (3)	0.75459 (13)	0.0517 (8)	
H8'A	1.1431	0.7112	0.7841	0.078*	
H8'B	1.1307	0.8011	0.7294	0.078*	
H8'C	1.0486	0.6625	0.7341	0.078*	
C9'	0.8503 (3)	0.8648 (3)	0.73878 (11)	0.0361 (6)	
C10'	0.8695 (3)	0.8573 (3)	0.68070 (11)	0.0454 (7)	
H10B	0.9502	0.8189	0.6663	0.055*	
C11'	0.7697 (4)	0.9065 (4)	0.64424 (13)	0.0623 (10)	
H11B	0.7848	0.9015	0.6055	0.075*	
C12'	0.6498 (4)	0.9619 (4)	0.66362 (14)	0.0677 (11)	
H12B	0.5838	0.9950	0.6385	0.081*	
C13'	0.6274 (4)	0.9684 (4)	0.72079 (15)	0.0694 (10)	
H13B	0.5451	1.0050	0.7345	0.083*	
C14'	0.7266 (3)	0.9210 (3)	0.75817 (12)	0.0540 (9)	
H14B	0.7104	0.9267	0.7968	0.065*	

### Atomic displacement parameters (Å^2^)

**Table:**

	*U*^11^	*U*^22^	*U*^33^	*U*^12^	*U*^13^	*U*^23^
N1	0.0404 (13)	0.0275 (11)	0.0346 (11)	−0.0001 (11)	0.0091 (10)	−0.0041 (9)
C2	0.0325 (14)	0.0351 (14)	0.0297 (12)	−0.0011 (13)	0.0019 (12)	−0.0017 (12)
O2	0.0508 (12)	0.0422 (11)	0.0423 (10)	0.0002 (10)	0.0178 (10)	−0.0038 (9)
C3	0.0391 (15)	0.0313 (14)	0.0285 (13)	−0.0026 (13)	0.0019 (13)	0.0015 (11)
C4	0.0411 (15)	0.0299 (13)	0.0369 (14)	0.0002 (13)	0.0029 (14)	−0.0022 (12)
O4	0.0783 (15)	0.0321 (10)	0.0440 (11)	0.0044 (12)	0.0186 (12)	−0.0040 (9)
C5	0.0492 (17)	0.0355 (14)	0.0345 (13)	0.0030 (15)	0.0127 (13)	−0.0003 (12)
C6	0.0438 (15)	0.0335 (13)	0.0413 (13)	−0.0009 (15)	0.0131 (14)	0.0015 (12)
C7	0.0408 (16)	0.0304 (14)	0.0379 (15)	0.0023 (13)	0.0076 (13)	−0.0029 (12)
C8	0.069 (2)	0.0383 (16)	0.0498 (17)	0.0068 (17)	0.0001 (18)	0.0023 (14)
C9	0.0424 (16)	0.0387 (16)	0.0425 (15)	−0.0041 (15)	0.0104 (14)	−0.0084 (13)
C10	0.075 (2)	0.055 (2)	0.067 (2)	−0.022 (2)	0.007 (2)	−0.0112 (18)
C11	0.090 (3)	0.079 (3)	0.112 (3)	−0.046 (3)	0.012 (3)	−0.025 (3)
C12	0.061 (3)	0.115 (4)	0.105 (3)	−0.021 (3)	−0.015 (3)	−0.047 (3)
C13	0.064 (2)	0.100 (3)	0.069 (2)	0.016 (3)	−0.022 (2)	−0.023 (2)
C14	0.060 (2)	0.0543 (19)	0.0529 (18)	0.0013 (19)	−0.0122 (17)	−0.0087 (16)
N1'	0.0458 (13)	0.0324 (12)	0.0283 (10)	−0.0111 (11)	−0.0044 (10)	0.0032 (9)
C2'	0.0389 (14)	0.0290 (13)	0.0341 (13)	−0.0029 (14)	−0.0037 (12)	−0.0004 (12)
O2'	0.0703 (14)	0.0451 (12)	0.0385 (10)	−0.0271 (12)	−0.0081 (11)	−0.0027 (9)
C3'	0.0493 (17)	0.0367 (15)	0.0251 (12)	−0.0032 (15)	−0.0030 (13)	−0.0004 (11)
C4'	0.0361 (15)	0.0295 (13)	0.0308 (13)	0.0006 (13)	0.0040 (12)	−0.0010 (11)
C5'	0.0483 (17)	0.0441 (16)	0.0364 (13)	−0.0171 (16)	−0.0055 (14)	0.0013 (13)
C6'	0.0631 (19)	0.0415 (15)	0.0290 (13)	−0.0164 (17)	−0.0049 (14)	−0.0005 (13)
C7'	0.0437 (16)	0.0348 (14)	0.0354 (14)	−0.0078 (14)	0.0014 (13)	0.0067 (12)
C8'	0.0450 (16)	0.0530 (18)	0.0570 (17)	0.0050 (17)	0.0057 (15)	0.0153 (16)
C9'	0.0420 (15)	0.0326 (14)	0.0336 (13)	−0.0035 (14)	0.0037 (13)	0.0026 (12)
C10'	0.0488 (17)	0.0521 (18)	0.0354 (13)	0.0087 (16)	0.0085 (15)	0.0035 (13)
C11'	0.072 (2)	0.080 (2)	0.0351 (15)	0.010 (2)	−0.0004 (17)	0.0130 (17)
C12'	0.066 (2)	0.083 (3)	0.054 (2)	0.022 (2)	−0.007 (2)	0.0172 (19)
C13'	0.052 (2)	0.086 (3)	0.070 (2)	0.023 (2)	0.0059 (19)	0.006 (2)
C14'	0.0559 (19)	0.066 (2)	0.0401 (15)	0.0104 (19)	0.0106 (16)	0.0035 (15)

### Geometric parameters (Å, º)

**Table:**

N1—C2	1.363 (3)	C14—H14A	0.9300
N1—C6	1.463 (3)	N1'—C2'	1.348 (3)
N1—C7	1.482 (3)	N1'—C6'	1.464 (3)
C2—O2	1.241 (3)	N1'—C7'	1.478 (3)
C2—C3	1.478 (4)	C2'—O2'	1.240 (3)
C3—C4	1.346 (4)	C2'—C3'	1.469 (4)
C3—C4'	1.488 (3)	C3'—C4'	1.334 (4)
C4—O4	1.339 (3)	C3'—H3'A	0.9300
C4—C5	1.495 (4)	C4'—C5'	1.501 (4)
O4—H4	0.97 (4)	C5'—C6'	1.482 (4)
C5—C6	1.515 (4)	C5'—H5'A	0.9700
C5—H5A	0.9700	C5'—H5'B	0.9700
C5—H5B	0.9700	C6'—H6'A	0.9700
C6—H6A	0.9700	C6'—H6'B	0.9700
C6—H6B	0.9700	C7'—C9'	1.517 (4)
C7—C8	1.523 (4)	C7'—C8'	1.527 (4)
C7—C9	1.524 (4)	C7'—H7'A	0.9800
C7—H7A	0.9800	C8'—H8'A	0.9600
C8—H8A	0.9600	C8'—H8'B	0.9600
C8—H8B	0.9600	C8'—H8'C	0.9600
C8—H8C	0.9600	C9'—C10'	1.390 (4)
C9—C10	1.385 (5)	C9'—C14'	1.392 (4)
C9—C14	1.388 (4)	C10'—C11'	1.380 (4)
C10—C11	1.386 (6)	C10'—H10B	0.9300
C10—H10A	0.9300	C11'—C12'	1.357 (5)
C11—C12	1.359 (6)	C11'—H11B	0.9300
C11—H11A	0.9300	C12'—C13'	1.373 (5)
C12—C13	1.362 (6)	C12'—H12B	0.9300
C12—H12A	0.9300	C13'—C14'	1.384 (5)
C13—C14	1.381 (5)	C13'—H13B	0.9300
C13—H13A	0.9300	C14'—H14B	0.9300
			
C2—N1—C6	119.3 (2)	C2'—N1'—C6'	119.8 (2)
C2—N1—C7	119.1 (2)	C2'—N1'—C7'	120.4 (2)
C6—N1—C7	118.4 (2)	C6'—N1'—C7'	116.79 (19)
O2—C2—N1	121.9 (2)	O2'—C2'—N1'	121.2 (2)
O2—C2—C3	120.3 (2)	O2'—C2'—C3'	121.7 (2)
N1—C2—C3	117.8 (2)	N1'—C2'—C3'	117.1 (2)
C4—C3—C2	120.8 (2)	C4'—C3'—C2'	123.3 (2)
C4—C3—C4'	124.5 (2)	C4'—C3'—H3'A	118.3
C2—C3—C4'	114.6 (2)	C2'—C3'—H3'A	118.3
O4—C4—C3	120.8 (2)	C3'—C4'—C3	124.0 (2)
O4—C4—C5	119.1 (2)	C3'—C4'—C5'	117.8 (2)
C3—C4—C5	120.1 (2)	C3—C4'—C5'	118.2 (2)
C4—O4—H4	116 (2)	C6'—C5'—C4'	111.5 (2)
C4—C5—C6	109.9 (2)	C6'—C5'—H5'A	109.3
C4—C5—H5A	109.7	C4'—C5'—H5'A	109.3
C6—C5—H5A	109.7	C6'—C5'—H5'B	109.3
C4—C5—H5B	109.7	C4'—C5'—H5'B	109.3
C6—C5—H5B	109.7	H5'A—C5'—H5'B	108.0
H5A—C5—H5B	108.2	N1'—C6'—C5'	112.2 (2)
N1—C6—C5	110.8 (2)	N1'—C6'—H6'A	109.2
N1—C6—H6A	109.5	C5'—C6'—H6'A	109.2
C5—C6—H6A	109.5	N1'—C6'—H6'B	109.2
N1—C6—H6B	109.5	C5'—C6'—H6'B	109.2
C5—C6—H6B	109.5	H6'A—C6'—H6'B	107.9
H6A—C6—H6B	108.1	N1'—C7'—C9'	110.0 (2)
N1—C7—C8	110.8 (2)	N1'—C7'—C8'	109.7 (2)
N1—C7—C9	110.5 (2)	C9'—C7'—C8'	115.1 (2)
C8—C7—C9	115.2 (2)	N1'—C7'—H7'A	107.2
N1—C7—H7A	106.6	C9'—C7'—H7'A	107.2
C8—C7—H7A	106.6	C8'—C7'—H7'A	107.2
C9—C7—H7A	106.6	C7'—C8'—H8'A	109.5
C7—C8—H8A	109.5	C7'—C8'—H8'B	109.5
C7—C8—H8B	109.5	H8'A—C8'—H8'B	109.5
H8A—C8—H8B	109.5	C7'—C8'—H8'C	109.5
C7—C8—H8C	109.5	H8'A—C8'—H8'C	109.5
H8A—C8—H8C	109.5	H8'B—C8'—H8'C	109.5
H8B—C8—H8C	109.5	C10'—C9'—C14'	117.5 (3)
C10—C9—C14	117.4 (3)	C10'—C9'—C7'	122.4 (3)
C10—C9—C7	122.7 (3)	C14'—C9'—C7'	120.1 (2)
C14—C9—C7	119.8 (3)	C11'—C10'—C9'	120.5 (3)
C9—C10—C11	120.5 (4)	C11'—C10'—H10B	119.8
C9—C10—H10A	119.7	C9'—C10'—H10B	119.8
C11—C10—H10A	119.7	C12'—C11'—C10'	121.5 (3)
C12—C11—C10	120.6 (4)	C12'—C11'—H11B	119.3
C12—C11—H11A	119.7	C10'—C11'—H11B	119.3
C10—C11—H11A	119.7	C11'—C12'—C13'	119.1 (3)
C11—C12—C13	120.1 (4)	C11'—C12'—H12B	120.4
C11—C12—H12A	120.0	C13'—C12'—H12B	120.4
C13—C12—H12A	120.0	C12'—C13'—C14'	120.4 (3)
C12—C13—C14	119.8 (4)	C12'—C13'—H13B	119.8
C12—C13—H13A	120.1	C14'—C13'—H13B	119.8
C14—C13—H13A	120.1	C13'—C14'—C9'	121.0 (3)
C13—C14—C9	121.5 (4)	C13'—C14'—H14B	119.5
C13—C14—H14A	119.2	C9'—C14'—H14B	119.5
C9—C14—H14A	119.2		
			
C6—N1—C2—O2	−169.0 (2)	C6'—N1'—C2'—O2'	169.0 (3)
C7—N1—C2—O2	−9.4 (4)	C7'—N1'—C2'—O2'	9.0 (4)
C6—N1—C2—C3	12.4 (4)	C6'—N1'—C2'—C3'	−11.1 (4)
C7—N1—C2—C3	172.0 (2)	C7'—N1'—C2'—C3'	−171.1 (2)
O2—C2—C3—C4	−166.8 (3)	O2'—C2'—C3'—C4'	170.0 (3)
N1—C2—C3—C4	11.8 (4)	N1'—C2'—C3'—C4'	−10.0 (4)
O2—C2—C3—C4'	11.4 (4)	C2'—C3'—C4'—C3	−179.2 (3)
N1—C2—C3—C4'	−170.0 (2)	C2'—C3'—C4'—C5'	−1.1 (4)
C2—C3—C4—O4	177.4 (3)	C4—C3—C4'—C3'	59.3 (4)
C4'—C3—C4—O4	−0.5 (4)	C2—C3—C4'—C3'	−118.7 (3)
C2—C3—C4—C5	−0.8 (4)	C4—C3—C4'—C5'	−118.8 (3)
C4'—C3—C4—C5	−178.8 (3)	C2—C3—C4'—C5'	63.2 (3)
O4—C4—C5—C6	150.8 (3)	C3'—C4'—C5'—C6'	30.6 (4)
C3—C4—C5—C6	−30.9 (4)	C3—C4'—C5'—C6'	−151.1 (2)
C2—N1—C6—C5	−44.4 (3)	C2'—N1'—C6'—C5'	40.8 (4)
C7—N1—C6—C5	155.8 (2)	C7'—N1'—C6'—C5'	−158.4 (2)
C4—C5—C6—N1	51.5 (3)	C4'—C5'—C6'—N1'	−48.9 (3)
C2—N1—C7—C8	131.3 (3)	C2'—N1'—C7'—C9'	−135.9 (3)
C6—N1—C7—C8	−68.8 (3)	C6'—N1'—C7'—C9'	63.5 (3)
C2—N1—C7—C9	−99.8 (3)	C2'—N1'—C7'—C8'	96.5 (3)
C6—N1—C7—C9	60.1 (3)	C6'—N1'—C7'—C8'	−64.1 (3)
N1—C7—C9—C10	−140.3 (3)	N1'—C7'—C9'—C10'	−133.8 (3)
C8—C7—C9—C10	−13.8 (4)	C8'—C7'—C9'—C10'	−9.2 (4)
N1—C7—C9—C14	41.5 (3)	N1'—C7'—C9'—C14'	47.8 (3)
C8—C7—C9—C14	168.0 (3)	C8'—C7'—C9'—C14'	172.3 (3)
C14—C9—C10—C11	−1.3 (5)	C14'—C9'—C10'—C11'	1.1 (5)
C7—C9—C10—C11	−179.6 (3)	C7'—C9'—C10'—C11'	−177.3 (3)
C9—C10—C11—C12	0.5 (6)	C9'—C10'—C11'—C12'	−0.7 (5)
C10—C11—C12—C13	0.9 (7)	C10'—C11'—C12'—C13'	−0.3 (6)
C11—C12—C13—C14	−1.4 (7)	C11'—C12'—C13'—C14'	0.9 (6)
C12—C13—C14—C9	0.5 (5)	C12'—C13'—C14'—C9'	−0.5 (6)
C10—C9—C14—C13	0.8 (5)	C10'—C9'—C14'—C13'	−0.5 (5)
C7—C9—C14—C13	179.1 (3)	C7'—C9'—C14'—C13'	178.0 (3)

### Hydrogen-bond geometry (Å, º)

**Table:**

*D*—H···*A*	*D*—H	H···*A*	*D*···*A*	*D*—H···*A*
O4—H4···O2′^i^	0.97 (4)	1.67 (4)	2.637 (3)	177 (4)

Symmetry code: (i) *x*−1/2, −*y*+3/2, −*z*+2.
